# Bereaved parents’ experiences of research participation

**DOI:** 10.1186/s12904-018-0375-4

**Published:** 2018-11-07

**Authors:** Ashleigh E. Butler, Helen Hall, Beverley Copnell

**Affiliations:** 1 The Louis Dundas Centre for Children’s Palliative Care, University College London Great Ormond Street Institute of Child Health, London, UK; 2 School of Nursing and Midwifery, Monash University, Melbourne, Australia; 3 School of Nursing and Midwifery, La Trobe University, Melbourne, Australia

**Keywords:** Research, Bereavement, Parent, Child, Death, Experience

## Abstract

**Background:**

As understandings of the impacts of end-of-life experiences on parents’ grief and bereavement increase, so too does the inclusion of bereaved parents into research studies exploring these experiences. However, designing and obtaining approval for these studies can be difficult, as guidance derived from bereaved parents’ experiences of the research process are limited within the current literature.

**Methods:**

We aimed to explore bereaved parents’ experiences of research participation in a larger grounded theory study exploring experiences of the death of a child in the paediatric intensive care unit. Data were obtained during follow-up phone calls made to 19 bereaved parents, five of whom provided data from their spouse, 1 week after their participation in the study. Participants were asked to reflect on their experiences of research participation, with a focus on recruitment methods, timing of research contact, and the location of their interview. Parents’ responses were analysed using descriptive content analysis.

**Results:**

Our findings demonstrate that despite being emotionally difficult, parents’ overall experiences of research participation were positive. Parents preferred to be contacted initially via a letter, with an opt in approach viewed most favourably. Most commonly, participants preferred that research contact occurred within 12–24 months after their child’s death, with some suggesting contact after 6 months was also appropriate. Parents also preferred research interviews conducted in their own homes, though flexibility and parental choice was crucial.

**Conclusions:**

Findings from this study offer further insight to researchers and research review committees, to help ensure that future studies are conducted in a way that best meets the unique needs of bereaved parents participating in research.

## Background

Over the past few decades, research has begun to highlight the impacts of the end-of-life care experience on parental grief, bereavement, and coping [1–7]. Consequently, in order to gain insights into their experiences of end-of-life care across a variety of locations and healthcare climates, the rates of inclusion of bereaved parents in research studies has also risen. However, despite studies which demonstrate participation in research studies is usually not harmful for bereaved parents [8–13], anecdotal evidence suggests that designing and obtaining approval for such sensitive research projects can be challenging.

Ongoing assumptions that bereaved parents are especially vulnerable and need to be protected from the perceived ‘harms’ or ‘risks’ of research may cause research review committees to be more hesitant in approving bereavement research [8–10, 13]. The research review committee’s role in assessing bereavement research can be challenging, as there is little empirical evidence to guide them in the ‘best’ way that these studies should be conducted. To date, the voices and opinions of bereaved parents on how to conduct bereavement research have largely been absent from the literature, with only a very limited number of studies published [8, 9, 11–13]. Many of these studies have attempted to provide guidance on important considerations when including bereaved parents into research studies, such as preferred contact methods; however such guidance is typically provided as vague, non-specific recommendations. More specific guidance around recruitment methods, contact timeframes, and data collection locations, based on the opinions of bereaved parents themselves, is urgently needed to assist both researchers and research review committees in moving forwards in bereavement research. We aim to address this gap by specifically exploring bereaved parents’ opinions of the research process, covering their experiences of being recruited into a bereavement study, the timeframe between their child’s death and their participation, and the location of their research interview. By specifically exploring these three key areas of research participation, we hope to provide concrete guidance for both bereavement researchers and research review committees, so that future studies may best meet the needs and preferences of bereaved parents and facilitate their ongoing inclusion into paediatric end-of-life care research.

## Methods

### Study design

The Bereaved Paediatric Intensive Care Unit (PICU) Parent Study explored bereaved parents’ experiences of the death of their child in the PICU, and their subsequent follow-up care [14]. In order to check on the wellbeing of participants, follow-up phone calls were undertaken by the research team 1 week after their interview, with parents asked to reflect on their experiences of research participation.

### Setting and participants

Twenty-six bereaved parents from 18 families took part in The Bereaved PICU Parent Study. Parents were recruited using purposive and theoretical sampling from four Australian PICUs by social workers involved in routine bereavement follow-up or via mailed letters 6–48 months after their child’s death in 2015–2016. For the primary study they took part in audio-recorded, semi-structured interviews at a time and location of their choice, conducted by the first author, a PICU nurse with prior qualitative research experience who was unknown to the participants. Participants were also advised that they would be called by a member of the research team within 1 week of their interview to ensure their wellbeing and to discuss their experiences of participating in the study.

### Research process

Parents’ reflections on their participation in bereavement research and the research process were collected during follow-up phone calls, lasting approximately 15–30 min. Parents were contacted 1 week after their interview by A.B, primarily to check on their wellbeing. During these phone calls, we asked six open-ended questions to explore their experiences of the research process, as outlined in Table 1. Parents’ responses were recorded verbatim in writing by the interviewer and checked with parents during the phone call.

**Table 1:** Follow-up interview questions

1. How did you feel in the hours and days after your interview? 2. Could you tell me a little bit about why you preferred to do the interview at home/over the phone/at the university? 3. When we first contacted you about this study, it had been xx months/years since [insert child’s name] had died. Did you feel this was appropriate, or would there have been a better timeframe for us to contact you? 4. You were initially contacted through a letter from the research team/a letter from the hospital/your bereavement social worker. How did you find this process? 5. Was there anything about the letter that you received that you liked or didn’t like? 6. How did you find participating in this research study overall?	

### Data analysis

Data from the follow-up interviews were subjected to descriptive content analysis, informed by the processes described by S Elo, et al. [15]. Content analysis is used to explore common issues and experiences within a set of data from both a qualitative and quantitative perspective [15, 16]. It involves establishing categories which describe the data, and identifying the frequency with which they occur, proving both a qualitative overview of the key concerns for participants and an indication of how commonly they occurred [17].

Data analysis was primarily undertaken by the first author, in consultation with the research team. The ‘preparation’ phase of content analysis commenced by identifying each individual question within the follow-up interview as the units of analysis, and re-reading the data multiple times to facilitate familiarity. During the ‘organising’ phase, open coding was undertaken, with common codes within each question grouped into categories. Where possible, these categories were further grouped based on similarities, which allowed key concepts in each question to be described and explored. Frequencies of each code and category were also identified at this stage, and analysed using descriptive statistics.

### Ethics

This study was reviewed and approved by four human research ethics committees. Written informed consent was provided by all participants prior to their interview, with verbal consent reconfirmed at the commencement of each follow-up phone call. Participants were encouraged to rely on personal coping strategies during and after their interviews, with breaks from the interview process utilised as required. Social workers associated with the study were also available for follow-up care of participants if ongoing distress was noted during the interviews or follow-up calls. In order to protect participants’ privacy, all data have been de-identified, and pseudonyms are used for all participants and their children.

## Results

Twenty-four out of the 26 participants in The Bereaved PICU Parent Study provided data on their experiences of research participation during follow-up phone calls. Direct follow-up was undertaken with 19 parents, five of whom also provided comments from their spouse. Two parents had mentioned they may be unavailable for further contact after their interview, and were subsequently lost to follow-up. Characteristics of all participants and their children are provided in Table 2. During follow-up phone calls, parents were asked how they felt after participating in their interviews, and were asked to reflect on the timing between their child’s death and researcher contact, the method of contact and the location of their interview.

**Table 2:** Characteristics of participants and their children

Direct follow up	Child’s name	Child’s age	Cause of death	Illness type	Time since death
Layla	Lucas	Infant	Neurological injury	Chronic	7 months
Daniel	Olivia	Teenager	Metabolic condition	Chronic	8 months
Lucy	William	Toddler	Septic shock	Acute	1 year, 1 month
Alice	James	Toddler	Accident	Acute	1 year, 2 months
Emma	Charlotte	Infant	Sudden Infant Death Syndrome	Acute	1 year, 4 months
Evelyn	Henry	Infant	Congenital heart disease	Chronic	1 year, 6 months
Jasmine	Mason	Infant	Metabolic condition/Liver failure	Acute	1 year, 6 months
Zara	Noah	Teenager	Multi-organ dysfunction	Acute	1 year, 8 months
Abigail	Amelia	Infant	Congenital heart disease	Chronic	1 year, 10 months
Sarah & Connor	Sophie	Infant	Sudden Infant Death Syndrome	Acute	2 years
Isabelle	Ava	Teenager	Cardiac arrest	Acute	2 years, 10 month
Vicki & Nate	Emily	Infant	Sudden Infant Death Syndrome	Acute	2 years, 10 month
Piper & Edward	Ethan	Teenager	Neurological injury	Chronic	3 years
Charlie	Liam	Infant	Liver failure	Acute	3 years
Erin	Ruby	Toddler	Congenital heart disease	Chronic	3 years, 6 months
Eva	Thomas	Teenager	Anaphylaxis	Acute	3 years, 8 months
Comments relayed by spouse					
Hannah	Olivia	Teenager	Metabolic condition	Chronic	8 months
Hudson	William	Toddler	Septic shock	Acute	1 year, 1 month
Joshua	Henry	Infant	Congenital heart disease	Chronic	1 year, 6 months
Ryan	Noah	Teenager	Multi-organ dysfunction	Acute	1 year, 8 months
Zoe	Liam	Infant	Liver failure	Acute	3 years
Lost to follow up					
Jessica	Ella	Infant	Congenital heart disease	Chronic	2 years, 6 months
Imogen	Chloe	Toddler	Multi-organ dysfunction / Septic shock	Acute	4 years

### The timing of research contact

Initially, we contacted parents 6–18 months after the child’s death in the PICU. This time frame was chosen to avoid early intense grief, and to minimise the impacts of memory and recall bias on data collection. However, due to lower than anticipated recruitment within this timeframe, we extended an invitation to all bereaved parents up to 48 months after their child’s death.

At participation, most families were 12–24 months into their bereavement (See Table 2). This was also the most commonly preferred timeframe for research contact, both for parents contacted at 12–24 months and for four parents who were contacted later in their bereavement (See Table 3). Parents suggested that this timeframe ensured that they still remembered what happened, how they felt, and what they needed very clearly. Many parents also noted that talking about their experiences would always hurt, but felt that at 12 months, enough time had passed that the interview was not significantly painful for them.

**Table 3:** Actual and preferred research contact timeframes

Direct follow up	Contacted at:	Contact preference:
Layla	7 months	Prior to 12 months
Daniel & Hannah	8 months	Prior to 12 months
Lucy & Hudson	1 year, 1 month	12–24 months
Alice	1 year, 2 months	12–24 months
Emma	1 year, 4 months	12–24 months
Evelyn & Joshua	1 year, 6 months	12–24 months
Jasmine	1 year, 6 months	12–24 months
Zara & Ryan	1 year, 8 months	Prior to 12 months
Abigail	1 year, 10 months	12–24 months
Sarah & Connor	2 years	Around 12 months
Isabelle	2 years, 10 months	Around 12 months
Vicki & Nate	2 years, 10 months	2–3 years
Piper & Edward	3 years	3–4 years
Charlie & Zoe	3 years	No opinion
Erin	3 years, 6 months	3–4 years
Eva	3 years, 8 months	12–24 months

Though five parents specifically suggested avoiding contact within the first 12 months as “the pain might be too fresh” (Erin), this opinion was not shared by parents who actually took part before the 12-month anniversary of their child’s death (Layla, Hannah, and Daniel). These three parents all felt that waiting longer than 12 months may have impacted their ability to remember exactly how they felt and what they needed, and felt that talking about their experiences helped them realise what they currently wanted and needed for support, as none of these parents had received follow-up care from the hospital or other bereavement services. In addition, one couple (Zara and Ryan) who participated at 18 months also mentioned they would have preferred to be contacted before 12 months, as the interview offered them a chance to debrief after their child’s death that they had otherwise not received, as no follow-up care was provided for them.

For the 9 parents who took part more than 2 years after their child’s death, four parents felt this timeframe was “okay” (Eva) but would have preferred to take part earlier in their bereavement because they felt they would have remembered their experiences more clearly. The remaining parents were happy with the contact time frame, suggesting that it provided sufficient time to work through any issues they had experienced. Erin also suggested that opportunities to discuss her child with family and friends lessened over time, and noted that the interview offered a forum to talk about her child when there was otherwise “no space to talk about [her]” in daily life.

### The method of research contact

Due to differences in local site requirements, we used three methods to invite potential participants into the study. At hospital 1, we were able to obtain contact information for eligible families and send an invitation letter directly from the research team. Hospitals 2 and 3 requested that social workers approach eligible families to obtain permission for research letters to be sent from the research team. Invitation letters at Hospital 4 were sent from a hospital based research nurse on behalf of the research team, with an enclosed ‘consent to contact’ card for interested parents to return. All mailed letters were then followed up with a phone call 2 weeks later. Further detail of our recruitment procedures has been published elsewhere [18].

Overall, 17 out of the 19 parents who provided direct comments felt that letters were an appropriate form of initial contact with bereaved families, regardless of whether the letter came directly from the research team or from another hospital staff member. Only 1 parent suggested a phone call would have been preferred as the initial contact method (Layla), and one parent did not share an opinion. Parents commented that a letter was a sensitive way to invite them into research participation, as it gave them time to consider the research project and make an appropriate decision, rather than being “put on the spot” (Connor). Many parents also felt a ‘cold-call’, or an unsolicited phone call from a research team member, would be an invasion of privacy and would be too shocking to enable them to consider what was being asked. Three parents from hospital 4 did mention they were confused by the arrival of a letter from the hospital, particularly in the absence of any previous contact, and noted it was helpful to have the name of the researcher and the research study on the back of the envelope. Many parents from hospital 4 also commented favourably on the use of an ‘opt-in’ card they could fill in and return if they were interested in the study; they suggested that this method of contact left the decision to participate entirely up to them without any pressure from the research team or hospital, and required no effort if they did not want to take part.

Four out of five parents who were initially approached by social workers also provided comments during follow-up phone calls. These parents all felt that contact through social workers was appropriate, but noted that this was only because they already had ongoing personal relationships with the social work team through routine bereavement follow-up. A follow-up letter and phone call from the research team was still desired, as it gave parents time to recall what the social worker had mentioned during the initial phone call, think of questions for the research team, and make a decision.

### The location of the interview

Parents in our study were encouraged to choose an interview type (face to face or phone) and location that was comfortable and convenient for them. The vast majority of parents (20 parents from 12 families) preferred face to face interviews in their own homes. Parents commented that discussing their child’s death was easier in their own home environment as it provided comfort, reduced feelings of vulnerability, and offered sufficient privacy for emotional expression. In addition, the home environment contained positive memories of the deceased child; for some parents, these memories provided comfort, whilst others felt more able to share their child’s life with the interviewer. For these reasons, the family home was often considered a “safe place” (Edward). In contrast, nine parents specifically mentioned that returning to the hospital for interviews would be too difficult, as that was “where it [the child’s death] happened” (Edward). In addition, parents commented that returning to the hospital added many logistical challenges to an already difficult task, such as locating and paying for parking, and having to drive home afterwards.

Only one parent (Emma) chose to do a face to face interview in a location other than her home. At her request, Emma’s interview was conducted in a private office in one of the university buildings. At the time of her interview, Emma was living in shared accommodation, and did not feel her home offered adequate privacy. However, Emma was also uncomfortable returning to the hospital, and noted she would not have participated in the study if the only alternative location for her interview was at the hospital, reinforcing the need for flexibility in interview locations.

Five parents preferred to participate in phone interviews. All three of the parents who were followed up noted that it would have been “too hard to meet face-to-face” (Eva). These parents commented that they would have either felt uncomfortable expressing their emotions in the presence of a stranger, or felt that they would have been more emotional talking about their experiences face-to-face than over the phone. One mother appreciated that there were options and that she could choose what suited her best, stating that if the only option was a face-to-face interview, she would not have taken part because she felt it would have been too difficult to talk about her experiences in person.

### Overall experiences of research participation

Although comments such as ‘It was difficult’ and ‘it was painful to talk about’ were common, all 24 of the parents who provided feedback on their research experiences mentioned that they were pleased and thankful to have taken part. They saw having a forum to talk about their child as a benefit; Erin said “It was good to talk. I haven’t told the story in a long time”, while Layla expressed gratitude to the researchers: “Thank you for listening and taking the time to know his story.” Importantly, none of the parents who participated expressed any regret over taking part, nor any concerns about their experience. Instead, many commented that they had “got a lot out of it [the research interview]” (Charlie).

Overall, half of the parents who were followed-up mentioned that they experienced increased fatigue, and increased episodes of crying, numbness, or heightened emotional distress in the 24–48 h after their interviews. However, the parents did not view these emotions negatively; instead, most commented that the interview provided a sense of emotional release that allowed them to “get [things] off your chest” (Charlie) or “take the weight off your shoulders” (Zara). Evelyn said “It really hit home … but it was a good release” while Alice commented “I cried, but I’m not too upset. Overall, it’s a positive experience.” Many suggested that despite the difficulties, it was “better to talk about it” (Eva) and remember their experiences, both positive and negative. Three couples suggested that the interview provided a space for them to talk about their experiences together for the first time, whilst another mother appreciated the opportunity to have a conversation entirely focused on her child, suggesting that research participation may be highly valued by bereaved parents. This is supported by the fact that not all parents in our study noted significant emotional distress after their interviews. Twelve parents commented that they felt okay or even ‘good’ after their participation, suggesting that perceptions of bereaved parents as especially ‘vulnerable’ to emotional distress might not be accurate.

Finally, many bereaved parents shared their motivations for participation in the research study. For most, the desire to help others and improve the experiences of families in the future was the main influencing factor in their decision to take part. Some parents, like Evelyn, felt like they were “flying blind” in the PICU, with no idea of what would happen during or after the death, and no support available to them. As a result, many wanted to take part in the research study because “if some of the systems can change because of Henry and because of what we put into this research, all the better for it” (Evelyn). For some parents, participation in research also allowed them to keep their child’s memory alive, “connect[ing] us with our child” (Charlie) or “acknowledg[ing] her life” (Erin), or create something positive from what had often been an otherwise traumatic experience.

## Discussion

Despite a vast number of studies demonstrating that bereaved persons, particularly bereaved parents, find value in participating in research studies and talking about their experiences [8–13, 19], little is known about their experiences of the research process itself. Without this information, it is difficult to design recruitment strategies and identify appropriate locations for data collection that best facilitate the inclusion of bereaved families into research studies. As part of routine follow-up after participation in a bereavement study, we explored parental experiences of the research process, in order to shed light on their preferred recruitment timeframes, contact methods, and locations for data collection.

The majority of bereaved parents in our study felt that the most appropriate timeframe to contact them regarding research participation was 12–24 months after their child’s death, with five parents preferring contact from 6 months. In previous studies, researchers have attempted contact somewhere between 6 months to 4 years after death [9, 20–30], but there is little empirical research to support these decisions. Studies of parental grief trajectories indicate that the passage of time is only one factor in a complex process [6, 13, 31–33], and thus there may be no time period that is universally appropriate. Respecting parents’ autonomy by giving them the opportunity to participate if they wish to do so may be more important than attempting to find a timeframe that will be least painful [13]. Parents in our study also supported the timeframe of 12–18 months as being optimal for recall. This presents a very narrow window for researchers that, as in our study, may be impractical. Researchers may need to balance parental preferences with pragmatic considerations. Though more research exploring bereaved parents’ preferred research contact timeframes is urgently needed, we suggest researchers consider approaching families 6 months- 2 years after a child’s death.

It is important to note that none of the parents in our study who expressed a wish for research participation prior to 12 months after their child’s death received any follow-up care from the hospital or other bereavement services. It is possible that their views are reflective of a desire for follow up care [34], rather than actual research participation. This presents a potential ethical dilemma for researchers, who could be seen as exploiting parents’ vulnerability. Though we recommend that parents still be given an opportunity to participate in research at this stage, heightened caution may be required. In addition, whilst research participation may be therapeutic for families, it cannot replace appropriate bereavement support, which should be provided by the child’s hospital or by local bereavement services [34–36]. It is important that researchers do not attempt to assume a counselling role.

During follow-up phone calls, we also asked the bereaved parents to comment on the way they were initially contacted for research participation and whether they would have preferred a different contact method. Consistent with previous studies of bereaved parents [9, 10] and bereaved adults more generally [19], parents in this study typically preferred that initial contact be undertaken via a mailed letter, rather than from an unexpected phone call. This was particularly true for letters that utilised an ‘opt-in’ approach, which placed parents in control of any further contact with the research team. Bereaved adults have also shown a preference for initial research contact through hospital personnel [19], though our experiences have suggested that this preference largely depends on a positive ongoing or pre-existing relationship with the staff member making contact. As such, we suggest that initial research contact with bereaved parents occur via a mailed letter that includes a reply-paid ‘opt-in’ card (with the study name on the outside to provide some warning), which both promotes parental autonomy and minimises intrusion and distress as much as possible.

Consideration of the location of research interviews is also particularly important when conducting bereavement research. Many parents in our study expressed a strong aversion to returning to the hospital, citing an association with negative memories and heightened distress. Instead, bereaved parents strongly preferred to be interviewed in their own homes. This location typically provided appropriate privacy and comfort, was noted to be a “safe place” for parents, and minimised parental inconvenience. The preference for interviews in the family home is not unique to our study, and has also been expressed by bereaved parents and siblings in previous studies [9, 10, 12]. However, five parents in our study preferred to do their interviews over the phone and did not find this format caused additional distress, and one parent preferred to be interviewed at the university, demonstrating the importance of providing parents with choices over where their interview takes place. As such, we strongly recommend that flexibility is ingrained into the research protocol, with bereaved family members offered various options for where or how their interview takes place, including in their own homes, to ensure they feel as comfortable as possible.

Within the current literature, it is becoming increasingly apparent that though bereaved parents find research participation emotionally painful, they still describe it as a positive experience and are thankful to have taken part [8–10, 12]. Our findings add weight to this concept, further demonstrating the importance of facilitating the inclusion of bereaved parents into research studies. For most parents, including those in this study, research participation provides a valuable opportunity to talk about their child that can often be missing in their daily lives, helps preserve their bond with their child by keeping their memory alive, and may help them to feel their child’s life and death had meaning and would be helpful to other families in the future [8–10, 12, 13, 37]. These findings also offer valuable insights into societal views of childhood death and longer-term parental bereavement. The fact that less-recently bereaved parents value research participation as a place to talk about their child that they do not have in daily life suggests a societal expectation to ‘move on’ from grief and, in a sense, act as though the deceased child did not exist [37]. However, research demonstrates that bereaved parents never ‘get over’ their child’s death, and want to continue to incorporate their deceased child into their lives and talk about them no matter how much time has passed [38, 39]. Though a detailed discussion on ways to change societal perceptions of ‘acceptable’ parental bereavement is beyond the scope of this article, it is important for bereavement researchers to publicise these findings to the public as well as the academic community, in order to begin to ‘normalise’ parental bereavement experiences and needs.

Whilst including parents in bereavement research may provide some therapeutic benefits, it is also important that they are fully aware of all potential risks before deciding to take part. Aside from emotional distress during the interview, over half of the parents in our study reported ongoing heightened emotions and fatigue in the days following their interview. Though post-interview fatigue is briefly mentioned by both K Dyregrov [9] and JL Buckle, et al. [8], it has not been extensively discussed within the literature, nor noted as a consideration for bereavement research planning. Therefore, in addition to the likelihood of ongoing emotional distress, we suggest that the likelihood of increased fatigue in the 24–48 h following research participation be made explicit on participant information sheets. This way, bereaved parents are able to make informed decisions about research participation, and are empowered to schedule interviews so that they do not cause significant disruption to their lives.

### Strengths and limitations

Our study was strengthened by the inclusion of both mothers and fathers from four different hospitals, whose children suffered from a variety of acute and chronic illnesses and injuries, and who experienced varied follow-up care, increasing the transferability of our findings. Findings are also reported according to the Consolidated Criteria for Reporting Qualitative Research [40]. However, there are some limitations to our study.

Firstly, the opinions of bereaved parents provided are only from those who had agreed to participate in the study, and who may have been more inclined to view bereavement research favourably. The views of parents who had chosen not to take part are unknown, and as such it is unclear whether some aspect of the recruitment process or contact timeframe influenced their decision not to participate. Where possible, we did attempt to enquire about the reason for their decision not to take part. Though responses were limited, most parents cited a lack of time or the desire to ‘move on’ as the primary reason for not taking part, rather than any specific concerns about recruitment timing or methods.

The findings were also limited by the fact that not all parents were able to be followed up directly. Two parents were lost to follow up, and five parents had comments relayed by their spouses. It is possible that these parents had negative comments that were not expressed to the research team, or were misrepresented by their spouses. However, the comments provided were consistent with the broader comments offered and offer valuable insights into the parents’ experiences.

Finally, participant feedback during follow-up phone calls was solicited by the same researcher who met with the parents and undertook the research interviews. Some parents may not have felt comfortable expressing negative or critical opinions of the research process to someone they had already met, which may have led to a higher incidence of positive feedback. However, similarly to the findings of J Hynson, et al. [10], a significant number of participants also spontaneously expressed positive feedback on the research process during or after their recruitment or interview participation, which suggests that our method of soliciting feedback was not a major limitation to the trustworthiness of the findings. In future studies, it may be beneficial to adopt alternative methods of seeking feedback about the research process, such as a survey or through a non-interviewing member of the research team, to ensure parents feel free to express both positive and negative feedback without fear of offending the interviewer.

## Conclusion

Despite ongoing research demonstrating that bereaved parents may find research participation beneficial rather than harmful, it can be difficult to design and gain approval for such emotionally laden studies. In part, this difficulty is caused by a lack of empirical evidence that explores how and when to approach bereaved parents in a way that promotes autonomy and safety whilst minimising harm and distress. Though further research is needed, our findings begin to offer suggestions for appropriate research contact timeframes, contact methods, and interview locations. These suggestions can help researchers design studies which empower bereaved parents, and assist research review committees in evaluating research proposals to facilitate the ongoing inclusion of bereaved parents into studies which are best suited to their needs.

## Abbreviation

PICU: Paediatric Intensive Care Unit
